# Sequencing and variant calling of SARS-CoV-2 from floor swabs: a potential tool for identifying emergent lineages

**DOI:** 10.1099/mgen.0.001575

**Published:** 2025-12-19

**Authors:** Benazir Hodzic-Santor, Aaron Hinz, Rees Kassen, Ju-Ling Liu, Haig Djambazian, Sally Lee, Alexandra Hicks, Calvin Sjaarda, Henry Wong, Prameet M. Sheth, Caroline Nott, Derek R. MacFadden, Anne-Marie Roy, Jiannis Ragoussis, Lucas Castellani, Michael Fralick, Alex Wong

**Affiliations:** 1 Department of Medicine, University of Toronto, Toronto, ON, Canada; 2 Division of General Internal Medicine, Sinai Health System, Toronto, ON, Canada; 3 Department of Biology, Carleton University, Ottawa, ON, Canada; 4 Department of Biology, McGill University, Montreal, QC, Canada; 5 McGill Genome Centre, Victor Phillip Dahdaleh Institute of Genomic Medicine, McGill University, Montreal, QC, Canada; 6 Department of Human Genetics, McGill University, Montreal, QC, Canada; 7 Department of Biology, University of Ottawa, Ottawa, ON, Canada; 8 Division of Microbiology, Kingston Health Sciences Centre, Kingston, ON, Canada; 9 Department of Pathology and Molecular Medicine, Queen’s University, Kingston, ON, Canada; 10 Infectious Disease Sequencing Laboratory, Kingston Health Sciences Centre, Kingston, ON, Canada; 11 Ottawa Hospital Research Institute, Ottawa, ON, Canada; 12 Faculty of Medicine, University of Ottawa, Ottawa, ON, Canada; 13 Sault Area Hospital, Sault Ste. Marie, ON, Canada; 14 Clinical Sciences Division, Northern Ontario School of Medicine University, Sudbury, ON, Canada; 15 Department of Medicine, Sinai Health System, Toronto, ON, Canada

**Keywords:** built environment, environmental surveillance, viral diversity

## Abstract

Ongoing viral evolution is a key driver of global pandemics, such as COVID-19, contributing to the repeated emergence and spread of new variants of concern. Identifying emerging viral variants is crucial for controlling the spread of infection; however, patient testing is not always feasible, and clinical samples are not routinely sequenced. As a result, new approaches, such as environmental-based surveillance, are needed for monitoring genetic diversity. Floor swabs provide greater spatial resolution than other environmental sampling approaches, but pose challenges for genomic analyses due to microbial RNA/DNA yields. We investigate the potential of obtaining whole-genome diversity data from floor swab samples to detect circulating lineages of severe acute respiratory syndrome coronavirus 2 (SARS-CoV-2). Floor swabs (n=23) were collected and sequenced from public locations in Ottawa, Canada, during December 2022, and were compared with contemporaneous human samples. Low biomass recovery remained a challenge, as approximately half of the swabs did not yield sufficient genetic material for analysis. The most commonly identified lineages from the floor swabs were XBB, while B (12.5%) and BA (12.5%) lineages appeared less frequently. In contrast, swab results from humans most often identified BQ (49.3%), BA (23.8%) and BF (17.8%), with XBB detected at a lower prevalence (2.7%). XBB became the dominant lineage in the region in the month following floor swab collection, suggesting that floor swabs may offer early signals of emerging outbreaks in comparison with hospital-based clinical sampling. This may suggest a role for floor swabs in outbreak prediction; however, larger studies are needed to validate this approach.

**Impact Statement**

The emergence and spread of new variants in viral outbreaks and pandemics, such as COVID-19, underlie changes in disease severity and immune evasion. Identifying emerging viral variants is thus crucial for controlling viral outbreaks, but patient testing is not always feasible, as widespread screening in hospitals is not routinely performed and, in some settings, such as long-term care homes, access to testing can be limited. It is also important to highlight that sequencing of patient samples is not routinely performed because of the high associated costs, and the fact that most hospitals in many countries do not perform sequencing. We evaluated the potential for using environmental surveillance, and specifically floor swabbing, to detect circulating lineages of severe acute respiratory syndrome coronavirus 2 (SARS-CoV-2). While low biomass was a challenge, we successfully obtained diversity data from floor swabs. Our findings suggest that floor swabs may offer early signals of emerging outbreaks, although larger studies will be needed to validate this approach.

## Data Summary

Severe acute respiratory syndrome coronavirus 2 (SARS-CoV-2) sequence data are available in the European Nucleotide Archive (ENA) at the European Molecular Biology Laboratory - European Bioinformatics Institute (EMBL-EBI) under project accession number PRJEB91353. Sample accession numbers are given in Table S1 (available in the online Supplementary Material).

## Introduction

Mutations in the severe acute respiratory syndrome coronavirus 2 (SARS-CoV-2) genome have been linked to increased transmissibility and immune evasion [1,3], emphasizing the high value of identifying emerging viral variants as early as possible to control the spread of infection. As human testing has declined and, in some jurisdictions, disappeared [4], wastewater surveillance has emerged as a valuable tool for detecting and quantifying viral RNA levels across large urban areas, for predicting surges in cases and for monitoring viral variants [5,7]. However, wastewater surveillance primarily provides regional-scale data and is of limited use in assessing infection spread at an institutional level [8].

Multiple studies have shown that viral RNA can be detected on floors and other surfaces in the built environment [9,10], highlighting floor swabbing as a potential COVID-19 surveillance strategy with increased spatial resolution. Detection of SARS-CoV-2 on floor swabs has been associated with outbreaks in long-term care homes [11,12], hospitals [13] and university campuses [14]. Rising detection of viral RNA on floor swabs may even predate an outbreak [11], providing an opportunity for improved infection prevention and control measures. These strengths notwithstanding, low microbial RNA/DNA yields collected from the built environment may make it difficult to obtain whole-genome sequence information from floor swabs. It remains unclear whether viral variants can be accurately identified from genetic material collected on floor swabs, and how well these results represent the variants circulating in the general population.

We investigated the feasibility of extracting whole-genome diversity data from floor swabs to identify circulating variants of concern. To assess how these data reflect the predominant SARS-CoV-2 lineages in the general population, we also compared the floor results with contemporaneous human samples from the same geographic region.

## Methods

Environmental (floor) swabs were collected in December 2022 from libraries, schools and hospitals in Ottawa, Ontario, Canada; a description of the full swabbing study is given elsewhere [14], as is a description of the swabbing protocol [11,13]. Briefly, we used the P208 Environmental Surface Collection Prototype Kit (DNA Genotek) to swab ~2′ by 2′ areas of floor and stored the swabs in collection vials containing nucleic acid stabilization solution. An unused swab placed in a collection vial served as a negative processing control (NegCtrl1). Total nucleic acids were extracted from 23 environmental swab samples and the negative processing control using the MagMAX Viral/Pathogen II (MVP II) Nucleic Acid Isolation Kit (Thermo Fisher). SARS-CoV-2 RNA was detected by quantitative reverse transcriptase PCR (qPCR) amplification as described previously [11,12], and cycle thresholds (Cq) for each sample were recorded.

**Fig. 1.**
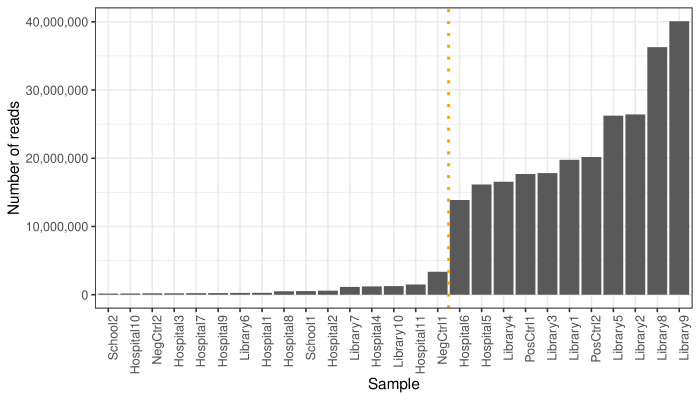
Reads after filtering, by sample. NegCtrl1 and NegCtrl2 are negative control swabs, and PosCtrl1 and PosCtrl2 are positive control swabs. A threshold of ten million reads was set, such that all samples with under ten million reads were excluded from variant analysis. The remaining samples are depicted to the right of the orange line.

Samples were sequenced at the McGill Genome Center by Illumina with the artic v5.3.2 workflow as described [15]. Three controls were included at the reverse transcription step for quality control of the library preparation and bioinformatic pipeline: NegCtrl2 (water), PosCtrl1 (a known viral isolate RNA from lineage B.1) and PosCtrl2 (synthetic SARS-CoV-2 reference RNA). A Python analysis pipeline was created based on the wastewater analysis pipeline by Sutcliffe *et al*. [16]. Reads were trimmed, and adapters and low-complexity sequences (<70 base pairs) were removed. To remove contaminants, sequences were aligned to a human reference genome (NCBI GCA_000001405.15), then to the SARS-CoV-2 reference genome (NCBI MN908947.3). Primer sequences were trimmed using iVar [17]. Variant calling and lineage assignment were performed using the Freyja package [18]. At this stage, samples with a read number <10,000,000 were discarded, based on a clear separation of negative controls at this read depth (Fig. 1; mean read number, included samples = 16,218,828.2; mean read number, excluded samples = 297,486.2). The sequence data for this study have been deposited in the European Nucleotide Archive (ENA) at the European Molecular Biology Laboratory - European Bioinformatics Institute (EMBL-EBI) under accession number PRJEB91353.

Variants called from floor samples were compared with variants from a patient cohort of 365 patients in Ottawa, Ontario. The sequences are publicly available at GISAID (https://gisaid.org/) and were collected as part of routine surveillance by the Eastern Ontario Regional Laboratory Association (EORLA). Samples from this cohort were collected from nasopharyngeal swabs between 1 December 2022 and 31 December 2022, and variants were called using the pipeline developed above. Finally, publicly available data from Public Health Ontario on variant prevalence were accessed and compared with the floor swab data. The Ontario COVID-19 Genomics Network (OCGN) performs whole-genome sequencing on samples received for diagnostic testing and assigns a Pango lineage [19]. These data show the total number of patient cases by lineage across Ontario in 2022–2023.

## Results

Twenty-three floor swabs were identified with detectable SARS-CoV-2 viral RNA at a cycle threshold of <40 [20,21]. After discarding samples with a post-filtering read number of <10,000,000, nine floor samples and the two positive controls remained. Negative controls were appropriately excluded through this process (Fig. 1). There was no significant difference in SARS-CoV-2 qPCR Cq values between swabs with low read numbers (mean: 32.6) and those with high read numbers (mean: 36.1) [t(17.7)=1.3, *P*=0.12, confidence interval (CI): −2.0 to 8.9; Wilcoxon rank-sum test, W=79, *P*=0.24]; however, the sample size was relatively small. Across all nine included samples, genome coverage was variable, with a median of 55.4% (CI: 32.0–88.0).

**Fig. 2.**
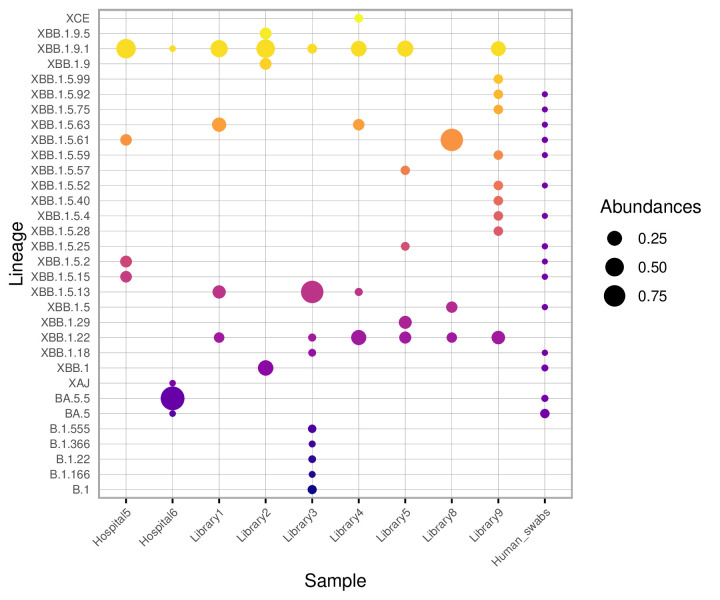
SARS-CoV-2 variant sub-lineages identified on floor swabs and in the human cohort. SARS-CoV-2 variant sub-lineages were identified from floor swabs collected at various locations. Sub-lineages detected in the human cohort are shown on the far right. The relative abundance of each variant in each swab is represented by the size of the points.

The most commonly identified lineages from the floor swabs were XBB, which were reported on all nine swabs, as shown in Fig. 2. The primary lineages identified from the human swabs were BQ (49.3%), BA (23.8%) and BF (17.8%). XBB was also present at this time and was reported in ten out of the 365 human clinical samples (2.7%).

As reported by Ontario Public Health, the primary variants identified at the end of December 2022 (weeks 49–51) were Omicron sub-lineages BQ.1.1, BQ.1 and BF.7. XBB.1.5 was circulating at this time, although with lower prevalence. By the week of 22 January 2023, XBB.1.5 was among the most prevalent lineages, becoming the dominant lineage by February 2023 [22]. All of the variants identified in Fig. 2 were reported between 1 January 2023 and 19 August 2023 [19]. During this time, 15,811 cases of XBB.1.5 and 1,871 cases of XBB.1.9 were recorded out of the 42,429 cases reported by the Ontario Health Data Platform.

## Discussion

This study demonstrates the feasibility of genome sequencing to identify SARS-CoV-2 variants from genetic material obtained through floor swabs. The most common variants identified on floor swabs were XBB.1.5 and XBB.1.9. At the time of the study, the XBB.1.5 lineage was the variant with the highest growth rate in the province [19,22], becoming the dominant variant by the end of January 2023 [22]. This aligns with studies showing that floor swabs can anticipate outbreaks [11], and wastewater data detecting emerging SARS-CoV-2 variants up to 12 weeks before public health reported a clinical detection [23].

XBB.1.5 was identified in human samples from the same time period (December 2022), though only in ~3% of samples. BQ and BF were not identified on floor swabs despite being the most prevalent lineages identified in the human cohort. This discrepancy may be related to sampling practices and government guidance at the time. Only 365 human samples were analysed in the month of December, a relatively small number compared to the over 5,000 COVID-19 cases reported in Ottawa during the week of 19 December 2022 alone [24]. Government guidelines limited PCR testing and sequencing to high-risk individuals and those in healthcare settings, while most people were advised to use rapid antigen tests at home and self-isolate if positive [19]. As a result, many milder SARS-CoV-2 infections, particularly those not requiring hospitalization, were not captured by PCR testing and were missing from public health data. The PCR-tested patient samples came from individuals with severe illness, while our floor swabs were collected in public areas, where people were generally healthier or in the early stages of infection. This difference in sampling creates a selection bias and may also explain why we observed high levels of XBB.1.5 in environmental swabs weeks before it was reflected in the public health case data. Indeed, the value of floor swabs lies in their ability to detect variants circulating in the broader population, variants that may be missed when sequencing is limited to the most severely ill patients.

Our study has several limitations. First, the floor samples were taken from public places, and we cannot know the viral characteristics of specific individuals that occupied those spaces over time. While we analysed the data from swabs taken from patients in the Ottawa region, how well these samples represent the true case distribution in Ottawa is unknown. Second, low biomass availability remained an issue, with only nine of the 23 floor swabs (excluding controls) yielding enough genetic material to accurately identify viral variants. We expect that biomass recovery will be a function of several factors, including rates of deposition (i.e. how many sick individuals occupy a given space and for how long) and persistence of viral particles (live SARS-CoV-2 can persist for days on solid surfaces) [13,25]. The frequency and details of cleaning regimens may also impact biomass recovery; in the hospitals swabbed here, floors are cleaned once daily. In a previous study [11], we found that SARS-CoV-2 qPCR signal correlated with COVID-19 cases at two different hospitals using different cleaning products, suggesting that floor swabbing results are robust across different cleaning protocols.

There are hundreds of identified SARS-CoV-2 sub-lineages, each reflecting minor genetic variations [26]. For example, sub-sublineages such as XBB.1.5.1 and XBB.1.5.2 typically involve SNPs without known functional impact. Given the variable genome coverage from floor swabs, the ability to accurately distinguish variants on such a fine scale remains unclear. Future studies should address whether increased recovery of biomass from floor swabs can lead to improvements in sequencing success rate and/or genome coverage. To fully understand the utility of floor swab sequencing will require studies in controlled environments where all individuals in the environment are concurrently tested.

## Conclusions

This study shows that whole-genome diversity data can be obtained from floor swabs. Low biomass availability remained an ongoing challenge, with about half the swabs failing to generate sufficient genetic material for analysis. Viral variants were detected on floor swabs prior to clinical surveillance, suggesting their potential utility in outbreak prediction, though larger studies are needed to validate this approach.

## Supplementary material

10.1099/mgen.0.001575 Table S1.

## Abbreviations

CI: confidence interval
EMBL-EBI: European Molecular Biology Laboratory - European Bioinformatics Institute
ENA: European Nucleotide Archive
EORLA: Eastern Ontario Regional Laboratory Association
OCGN: Ontario COVID-19 Genomics Network
qPCR: quantitative PCR
SARS-CoV-2: severe acute respiratory syndrome coronavirus 2
